# A Systematic Review of the Progression of Cutaneous Lupus to Systemic Lupus Erythematosus

**DOI:** 10.3389/fimmu.2022.866319

**Published:** 2022-03-11

**Authors:** Paul Curtiss, Amanda M. Walker, Benjamin F. Chong

**Affiliations:** Department of Dermatology, University of Texas Southwestern Medical Center, Dallas, TX, United States

**Keywords:** cutaneous lupus erythematosus (CLE), systemic lupus erythematosus, systematic review, autoimmunity, progression

## Abstract

Lupus erythematosus is an autoimmune disease that may manifest in a variety of organs and tissues including the skin, kidney, brain, heart and lung. Many patients present with cutaneous lupus, where disease is often limited to the skin, but are at risk for developing systemic lupus. The objective of our present study is to perform a systematic review of studies that investigated patient cohorts and populations for the occurrence of cutaneous lupus progressing to systemic lupus. Inclusion criteria required that studies present longitudinal data of patients with limited cutaneous lupus erythematosus who were followed for development of systemic lupus erythematosus. Studies were excluded if patients had concurrent diagnosis of SLE, or if they failed to present longitudinal data. Medline and Embase were searched for English language studies using the Ovid platform. A total of 25 adult studies were identified, as well as 8 pediatric studies. The rate of cutaneous to systemic lupus progression ranged between 0% to 42% in the adult studies and 0% to 31% in the pediatric groups. The variability in these rates were due to differences in patient populations, study design, criteria used to diagnose systemic lupus, and follow-up time. Common risk factors associated with systemic lupus erythematosus development including having positive anti-nuclear antibodies, hematologic abnormalities, and higher number of lupus classification criteria at baseline. This study emphasizes the importance for providers to routinely monitor for systemic lupus in patients with cutaneous lupus.

## Introduction

Cutaneous lupus erythematosus (CLE) is an autoimmune skin disease with a wide range of clinical presentations. Several subtypes exist including acute cutaneous lupus (ACLE), subacute cutaneous lupus (SCLE), and chronic cutaneous lupus (CCLE), with the most common CCLE subtype being discoid lupus erythematosus (DLE). As early as 1872, Moritz Kaposi identified a characteristic subset of patients with DLE and found that while they may present with limited cutaneous disease, some may progress to systemic involvement (1). Systemic involvement can range from mild in severity, affecting only a single organ system, to potentially severe systemic involvement, affecting multiple organ systems.

Since then, several classification criteria, including the American Rheumatism Association (ARA) criteria, American College of Rheumatology (ACR) criteria, Systemic Lupus International Collaborating Clinics (SLICC) criteria, and the European League Against Rheumatism/American College of Rheumatology (EULAR/ACR) criteria, have been developed to help clinicians monitor for the progression of CLE to systemic lupus erythematosus (SLE) (2–5). Clinically, the risk of patients with isolated CLE developing SLE is an area of interest to both the dermatologist and rheumatologist, and CLE patients. Current screening recommendations suggest monitoring patients for various lab abnormalities and clinical symptoms included in the lupus classification criteria sets, including the development of hematological abnormalities, autoantibodies including anti-nuclear antibodies (ANA) and double-stranded DNA (dsDNA) antibodies, and signs of joint, kidney or neurologic involvement (6). Current standard of care involves checking CLE patients for systemic disease on presentation as well as interval assessments for the development of SLE (6, 7).

The phenomenon of CLE developing to SLE has been studied in a variety of settings and populations, with the rate of progression ranging from zero to over thirty percent (8–10). Notably, methodologies amongst studies have often differed with respect to the studied population, definitional criteria of SLE, length of follow up, and study design. Prior reviews aimed at summarizing these studies have been limited to narrative reviews, narrow timeframe, or confined to a single subtype of CLE (11, 12). In order to better summarize these data, we performed a systematic reviews of all studies that have investigated patient cohorts and populations for the occurrence of CLE progressing to SLE. The information gleaned from this systematic review will help equip providers with counseling these patients about their prognosis and direct the management of these patients to track disease progression.

## Methods

This systematic review was conducted using the Preferred Reporting Items for Systematic Reviews and Meta-Analysis (PRISMA) guidelines (13). The objective was to identify studies of patients with skin limited cutaneous lupus and the rates of development of systemic lupus to better examine how studies evaluate and characterize this transition. The primary outcome of interest was the proportion of patients with CLE who developed SLE. Inclusion criteria were that studies identified cohorts of patients with CLE without SLE initially. Studies were excluded if patients had concurrent presentation of CLE and SLE, or did not present longitudinal data (either retrospective or prospective) for the development of SLE.

English language literature was searched using the MEDLINE and Embase databases. Databases were searched from inception until the date of the search using the Ovid platform. Databases were searched for articles with keywords, titles, abstracts including cutaneous lupus or its subtypes (i.e. discoid lupus, lupus panniculitus, lupus profundus, bullous lupus, subacute cutaneous lupus, lupus tumidus) and systemic lupus. Two separate reviewers (P.C. and A.W.) independently appraised all studies meeting inclusion and exclusion criteria. Disagreements were discussed and consensus reached involving a third reviewer (B.F.C.) whenever appropriate. Full text articles were then screened for inclusion in the present study and reference lists of primary studies were searched for additional studies meeting inclusion criteria.

## Results

After removing duplicates in the OVID platform, a total of 2,842 titles and abstracts were screened for articles potentially meeting inclusion criteria. Of these, 85 full-text articles were selected for in-depth review with a total of 33 articles relevant articles identified meeting our inclusion criteria. This included 25 articles of adult CLE patients, and 8 pediatric CLE studies, which will be summarized in the following sections. A complete PRISMA flow chart is included in **Supplementary Figure 1** (13).

### Adult CLE

Studies looking at adult CLE patients reported a broad range of CLE to SLE progression. The rate of CLE to SLE progression ranged from 0 to 42 percent of CLE patients developing SLE (**Table 1**). The number of patients with CLE only and therefore eligible to progress varied widely amongst studies, ranging from small cohorts of only 5 patients to large, database studies of over 20,000 patients (18, 24, 30). DLE was the most commonly studied CLE subtype amongst all studies examined (20/25). SCLE was the second most commonly represented subtype (10/25). Notably, one study found that patients with SCLE had higher rates of progression than those with DLE (9). Most studies analyzed CLE patients from multiple subtypes. While several studies did report on various CLE subtypes other than DLE (e.g. lupus erythematosus panniculitis, lupus erythematosus tumidus), this accounted for a relatively small proportion of the overall data studied.

**Table 1:** Summary of results from adult cohort studies.

Author	Year	Total CLE Patients (n)	CLE to SLE n, (%)	Time to Progression	SLE Diagnostic Method
Aitmehdi et al. (14)	2021	14	1 (17)	NA	NA
Al-Saif et al. (15)	2012	56	6 (11.8)	10.5 months (mean)	NA
Baek et al. (16)	2020	27	27 (4.3)	1.53 years (mean)	ICD-10
Black et al. (17)	2021	93	10 (10.8) by SLICC, 15 (16.1) by ACR	7.8 years (SLICC, mean)	SLICC and ACR
Braunstein et al. (18)	2013	5	1 (20)	NA	ACR
Callen et al. (19)	1982	56	4 (6.5)	NA	NA
Casarrubias et al. (20)	2019	8	2 (25)	NA	NA
Chanprapaph et al. (21)	2021	42	4 (9.5)	5.6 months (median)	SLICC
Drenkard et al. (22)	2019	190	9 (5.3) at one year and 16 (12.3) at three years	NA	ACR
Durosaro et al. (23)	2009	156	19 (12.2)	8.2 years (mean)	ACR
Gronhagen et al. (9)	2011	828	107 (12.9)	NA	ICD-10
Hall et al. (24)	2017	20,878	4,715 (11)	12.8 months (mean)	ICD-9
Healy et al. (25)	1995	58	3 (5.2)		ARA
Kindle et al. (26)	2016	9	0 (0)	NA	ACR
Leibowitch et al. (27)	1981	42	4 (9.5)	NA	ARA
Millard et al. (28)	1979	92	6 (6.5)	NA	ARA
Ng et al. (29)	2002	10	1 (10)	NA	NA
Petersen et al. (30)	2018	1674	199 (11.9)	2.05 years (median)	ICD-10
Preti et al. (31)	2019	12	5 (42)	NA	SLICC
Rees et al. (32)	2015	1002	145 (14)	NA	Read Codes
Schiodt et al. (33)	1984	56	5 (8.9)	NA	ARA
Scott et al. (34)	1959	274	14 (5)	NA	NA
Wieczorek et al. (35)	2014	77	13 (17)	8.03 years (mean)	ACR
Wu et al. (36)	2018	25	6 (24)	NA	ACR
Xie et al. (37)	2020	17	5 (29.4)	NA	ACR

ACR, American College of Rheumatology; ARA, American Rheumatism Association; CLE, cutaneous lupus erythematosus; ICD-9, International Classification of Diseases, ninth revision; ICD-10, International Classification of Diseases, tenth revision; NA, not applicable; SLE, systemic lupus erythematosus; SLICC, Systemic Lupus International Collaborating Clinics.

Studies used several different metrics to define SLE. Most studies (7/25) used the 1982 ACR SLE criteria (18, 22, 23, 26, 35–37). Four studies pre-dated the development of the 1982 ACR criteria and used ARA criteria (25, 27, 28, 33). Two studies used the 2012 SLICC classification criteria (21, 31). None have employed the 2019 EULAR/ACR criteria. One study used more than one classification criteria set to compare rates of CLE to SLE progression. From a cohort of 93 patients with CLE, our group reported 10.8% developing SLE under the SLICC criteria and 16.1% under the ACR criteria, highlighting potential differences between criteria sets (17). Five adult studies used diagnostic codes for large data sets (9, 16, 24, 30, 32). Six studies did not specify a defined criteria set/methodology (14, 15, 19, 20, 29, 34).

The length of follow up was variable among studies. For instance, 11 out of 25 studies only reported a range of years from which records were reviewed instead of average follow-up time (9, 15, 20–23, 27, 30–33). Some studies chose to report a range of years from which records were obtained and a minimum length of follow up of 6 months (16, 17, 37). Other studies chose to report median or mean length of time to follow up, ranging from a median of 40 to 48 months or a mean of 16.7 months to 5.75 years (14, 19, 26, 29). In addition, some studies reported variable rates that were dependent on length of follow up. For instance, Gronhagen et al. reported that when follow up data for one year was analyzed, 9.7% of CLE patients developed SLE; when sufficient follow up data was available for 3 years, this shifted to 16.7% (9).

Heterogeneous data on risk factors for CLE to SLE progression and time to progression were available from a minority of studies. From the adult studies, the most common patient and clinical risk factors associated with SLE development included positive ANA (5/25), hematologic abnormalities (2/25), and number of classification criteria met at baseline (2/25) (15, 17, 21, 25, 28, 35). Studies often differed on significant risk factors. Al-Saif et al. reported that CLE patients who progressed to SLE had more sunlight exposure, were ANA positive, and had a positive dsDNA antibody. They also found that progression of disease was significantly correlated with an earlier age of onset (p=0.044). Our group identified baseline risk factors for disease progression under the SLICC criteria including positive ANA (p=0.02), SLICC immunologic criteria (p=0.002), and SLICC total criteria (p=0.007) (17). Other studies identified baseline risk factors including non-scarring alopecia and high initial ANA titer ≥1:320 (21), hematologic abnormalities and positive ANA (28), and mucocutaneous criteria, positive ANA, total number of ACR criteria, and generalized DLE (35). Time to progression was reported inconsistently among studies and ranged anywhere from a mean of 5.6 months to a median of 8.2 years for adult cohorts (21, 23). One study reported significantly different median time to progression for subtypes of CLE including 3.04 years for DLE, 1.65 years for SCLE, and 1.04 years for localized CLE (p=0.018) (30).

### Pediatric CLE

Eight studies looking at CLE to SLE progression amongst pediatric cohorts were found. Similar to the adult cohort studies, there was also a broad range of progression rates among pediatric populations, ranging from 0 to 31 percent of patients developing SLE (**Table 2**). However, the cohort size of patients with CLE and therefore eligible to progress to SLE was notably smaller than that of adult cohort studies, ranging from 10 to 276 total patients (41, 43). Similar to adult studies, DLE was the most commonly analyzed subtype representing over 60% of pediatric studies. Two studies examined a mixed cohort of multiple subtypes (8, 40). One small cohort study was dedicated to lupus erythematosus profundus (43).

**Table 2:** Summary of results from pediatric cohort studies.

Author	Year	Total CLE Patients (n)	CLE to SLE (n, %)	Time to Progression	SLE Diagnostic Method
Arkin et al. (38)	2015	34	9 (26)	NA	ACR
Cherif et al. (39)	2003	16	0 (0)	NA	NA
Dickey et al. (40)	2013	38	1 (2.6)	NA	NA
Ezeh et al. (41)	2019	276	55 (20) by ACR and 69 (25) by SLICC	NA	ACR and SLICC
George et al. (10)	1993	16	5 (31)	NA	NA
Lee et al. (8)	2019	11	0 (0)	NA	ACR
Moises Alfaro et al. (42)	2003	27	7 (26)	NA	ACR
Tinoco-Fragoso et al. (43)	2016	10	0 (0)	NA	NA

ACR, American College of Rheumatology; CLE, cutaneous lupus erythematosus; NA, not applicable; SLE, systemic lupus erythematosus; SLICC, Systemic Lupus International Collaborating Clinics.

In terms of criteria sets for SLE diagnosis, pediatric studies most commonly used the ACR criteria to define SLE progression (3/8 studies) (8, 38, 42). Ezeh et al. reported rates of progression for both ACR (20%) and SLICC (25%) criteria in the same cohort of patients (41). The remainder of pediatric studies did not specify a specific classification or diagnostic criteria used to determine the progression of CLE to SLE in their patient cohorts (10, 39, 40, 43). Like adult studies, follow-up length for pediatric cohorts was variably reported, with studies reporting a median follow up time ranging between 1 and 11 years (8, 10).

Only three studies commented on risk factors for progression. Risk factors included: higher age at diagnosis of DLE and positive autoantibodies, positive serologies and higher-titer ANA, and positive family history for rheumatic disease (p<0.05) (38, 41, 42). Only one study, Arkin et al., reported data on time to progression and noted that pediatric patients were at greatest risk for CLE to SLE progression within the first year after CLE diagnosis (38). However, they note that their study was limited to a follow-up duration of 5 years.

## Discussion

This systematic review encompassed a broad range of studies, reporting on both adult and pediatric CLE groups. In adults, all but one study showed a proportion of CLE patients ultimately developing SLE. While a minority of CLE patients will go on to develop SLE, this proportion is sizeable enough to highlight the need for CLE patients to have ongoing monitoring for the development of SLE. Interestingly, data was somewhat more bimodal in the pediatric studies, with several studies reporting that no CLE patients progressing to SLE, but other studies reporting higher risk of 20%-30%. This discrepancy in reported risks may reflect study level characteristics or varying patient populations. The relatively limited number of pediatric studies highlights the need for more data to better characterize the risk of developing SLE within the pediatric population.

Studies used a variety of different metrics to define SLE. Larger population studies used diagnostic codes to identify patients with SLE. While this may be less rigorous on a patient level basis, it does allow for examining a significantly broader segment of the population and provide greater context of this phenomenon. For smaller studies, specific SLE classification criteria, including the ARA, ACR, and SLICC criteria, were employed for each patient and their disease course. Studies that examined multiple diagnostic criteria both supported the risk of transition to SLE. The similarly reported rates within studies that employed multiple SLE diagnostic criteria suggests that this distinction may not account greatly for the discrepancies in progression rates between studies. For example, Ezeh et al. reported on both SLICC and ACR criteria, yielding 20% progression under ACR criteria and 25% under SLICC criteria (41). Conversely, Black et al. reported 10.8% development from CLE to SLE using SLICC criteria and 16.1% with ACR criteria (17). The small variation in rates were thought to be, in part due to application of photosensitivity as a diagnostic criteria in ACR but not SLICC.

A variety of risk factors have been proposed to influence the risk of development of SLE, which was more commonly studied in adult CLE patients than pediatric CLE patients. Disease severity, CLE subtype, autoantibodies (anti-dsDNA and anti-Smith), arthritis, and high titers of ANAs have been reported to be more commonly found in CLE patients progressing to SLE than those who have not (11, 44). In our review of prior studies, the most common risk factor reported was a positive ANA (15, 17, 21, 28, 35, 41). Other common risk factors included hematologic abnormalities, age at CLE onset, lupus specific antibodies like dsDNA, and mucocutaneous criteria (15, 21, 25, 28, 35, 38, 41). Disparities in risk factor reporting can be attributed to differences in study design, population, and methods of reporting SLE diagnosis. Future larger-scale studies with uniform SLE diagnosis reporting are needed to further confirm risk factors that portend higher chance for systemic progression in CLE patients. In addition, most CLE patients who ultimately progressed to SLE in the studies examined by this review rarely met criteria that would signify involvement of major organ systems (e.g. renal, neuro), highlighting the overall mild severity of systemic involvement seen in CLE patients who progress to SLE (17, 21, 35).

It has been hypothesized that antimalarial treatment with may slow or prevent the progression of systemic disease (45). To address this hypothesis, there is an ongoing multi-center randomized controlled trial looking at whether hydroxychloroquine can halt progression of lupus in at-risk individuals such as those with CLE (46). Given that lupus medications may slow development to SLE, the rate of progression may be higher in untreated CLE individuals. While none of the reported studies looked at effects of therapies on progression, we hypothesize that because most patients in these studies were under treatment, reported rates of progression from CLE to SLE may be conservative.

In conclusion, this study summarized findings from adult and pediatric CLE patient groups showing ranges of progression to SLE. Prior studies showing up to 42% of CLE patients progressing to SLE highlight the importance for monitoring CLE patients for the development of systemic disease clinically at routine intervals. We recommend that providers perform complete review of systems to identify any new systemic symptoms such as small joint pains, and thorough skin exams to check for worsening skin disease and presence of oral ulcers lasting more than two weeks. Laboratory tests including ANAs and complete blood counts can be also ordered, with positive ANA titers being followed up with additional autoantibody tests including dsDNA and extractable nuclear antibody tests (6). Importantly, larger multi-center studies using standard and uniform reporting of SLE diagnosis and heterogeneous populations are necessary to better estimate rates of and identify risk factors for development of SLE in CLE patients.

## Author Contributions

PC, AW, and BC contributed to conception and design of the study. PC and AW contributed to the acquisition and analysis of the data. PC and AW drafted the manuscript. All authors contributed to manuscript revision, read, and approved the submitted version.

## Conflict of Interest

BC is an investigator for Daavlin Corporation and Biogen Incorporated and Pfizer Incorporated. He is a consultant for Bristol Meyers Squibb, EMD Serono, Horizon Therapeutics, and Biogen Incorporated.

The remaining authors declare that the research was conducted in the absence of any commercial or financial relationships that could be construed as a potential conflict of interest.

## Publisher’s Note

All claims expressed in this article are solely those of the authors and do not necessarily represent those of their affiliated organizations, or those of the publisher, the editors and the reviewers. Any product that may be evaluated in this article, or claim that may be made by its manufacturer, is not guaranteed or endorsed by the publisher.

## Supplementary Material

Supplementary Figure 1 PRISMA Flow Diagram for literature search. Diagram shows searching and selection strategy at each stage of search.
