# Supplementary figures and images for: A Systematic Review of the Progression of Cutaneous Lupus to Systemic Lupus Erythematosus
Source: Front Immunol. 2022 Mar 11;13:866319. doi: 10.3389/fimmu.2022.866319 (PMC8963103; doi:10.3389/fimmu.2022.866319)

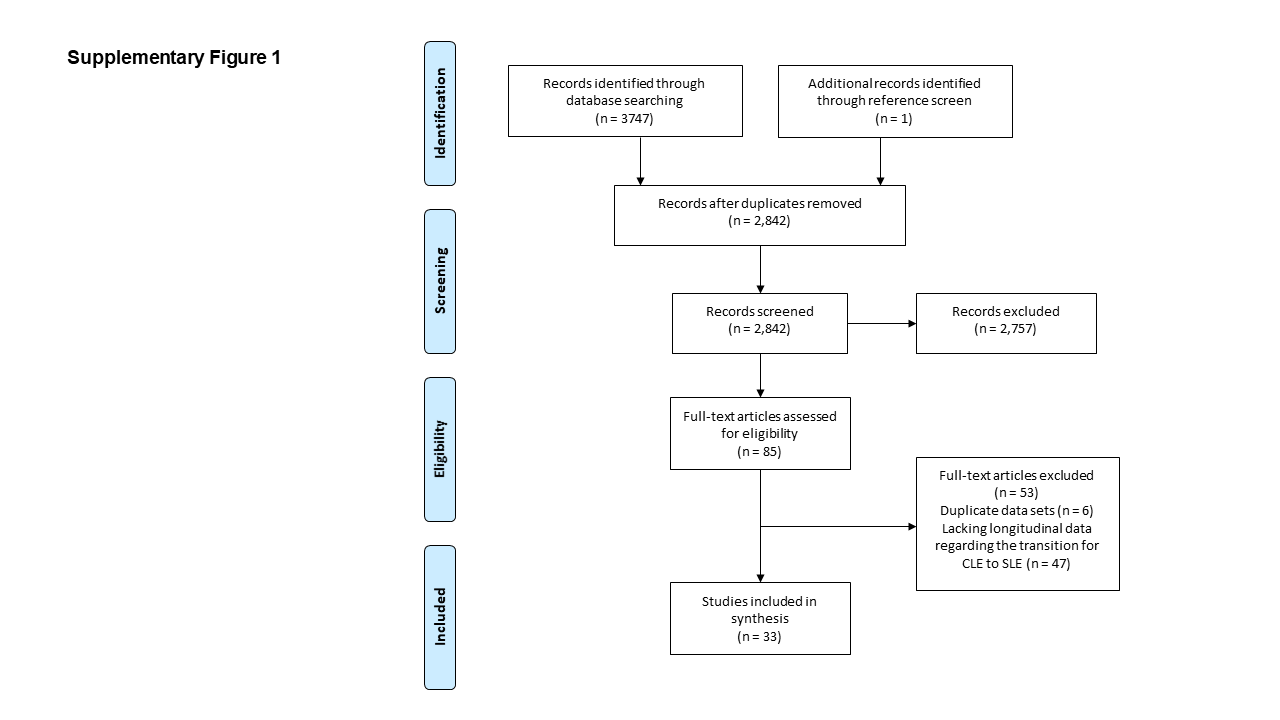

Supplement: Supplementary Figure 1 — PRISMA Flow Diagram for literature search. Diagram shows searching and selection strategy at each stage of search. [file Image_1.tif]
